# Spontaneous Perforation of Meckel’s Diverticulum in a Neonate

**Published:** 2012-01-01

**Authors:** Muhammad Qasim, Mahmood Shaukat

**Affiliations:** Department of Pediatric surgery, Mayo Hospital Lahore, Pakistan

**Dear Sir,**

Meckel’s diverticulum was first described in 1598 by Hildanus. Meckel’s diverticulum is said to be present in 2 % of population. It is twice as common in males as opposed to females. Most of the symptomatic cases present within the first 2 years of life. It may get inflame or perforate [1,2]. Its presentation as perforation is rarely seen in neonatal period.

A 7-day-old neonate weighing 2.5kg was referred to our department with abdominal distention for four days and absolute constipation for 2 days. The baby was born by C-Section at full term and was given rose water and few sips of cow’s milk after birth. He passed meconium normally. The baby started bilious vomiting on 2nd day of life followed by abdominal distention on the next day. On 7th day the abdomen became tense. On clinical examination the patient was tachypneic and the abdomen was tense and distended. Bowel sounds were absent. Rectum was empty on rectal examination. The laboratory investigations showed a deranged clotting profile. An erect abdominal x-ray was advised for the suspicion of a perforation, which showed free gas under the diaphragm.

A peritoneal drain was passed while the child was optimized for surgery. After normalizing the coagulation profile, surgery was performed. Intra-operatively the small bowel was adherent with inflammatory flakes all over it. A perforated Meckel’s diverticulum was found after mobilization of the gut (Fig. 1). The Meckel’s diverticulum with a small ileal segment was resected and an end to end anastomosis was performed. A pelvic drain was also placed. The immediate recovery was uneventful; however, the baby developed sepsis on 6th postoperative day and succumbed.

**Figure:**
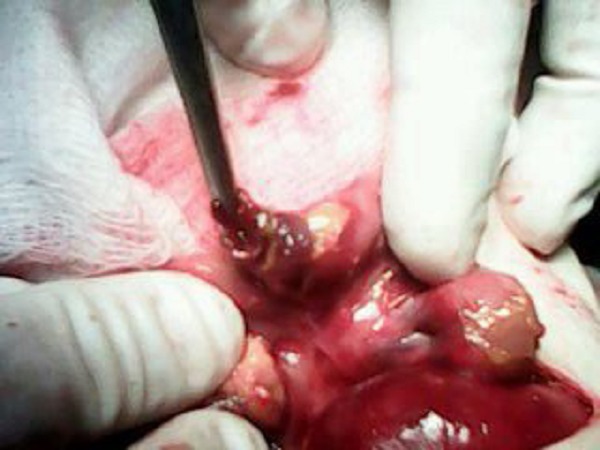
Figure 1: Perforated Meckel's Diverticulum

About 3-4% of Meckel’s diverticulae become symptomatic and perforation occurs in 10% of symptomatic cases. Pneumoperitoneum in a neonate is a serious entity and most of times indicate bowel perforation. In neonates the etiology of pneumoperitoneum is necrotizing enterocolitis, mechanical obstruction like atresia and stenosis, meconium ileus, hirschsprung’s disease, anorectal malformations etc. or it can be a spontaneous perforation. Meckel’s diverticulum perforation presenting with pneumoperitoneum is seldom reported in literature [2].

The etiology of perforation of Meckel’s diverticulum as described by various authors are acute inflammation with ectopic pancreatic or gastric mucosa, acute inflammation without any ectopic gastric or pancreatic mucosa, congenital focal muscular defect of diverticulum, narrow lumen with poor self emptying, and spontaneous perforation [2,3].

In our case the baby was full term and otherwise healthy. There was no history of perinatal distress or asphyxia. The histopathology did not reveal ectopic gastric or pancreatic mucosa; therefore the likely cause of perforation in our case could be spontaneous.

In conclusion, despite the rarity of the perforated Meckel’s diverticulum in neonates, it should be considered as one of the differential diagnosis of acute abdomen and pneumoperitoneum.

## Footnotes

**Source of Support:** Nil

**Conflict of Interest:** None declared

